# Epigenetic Regulation (Including Micro-RNAs, DNA Methylation and Histone Modifications) of Rheumatoid Arthritis: A Systematic Review

**DOI:** 10.3390/ijms222212170

**Published:** 2021-11-10

**Authors:** Melissa Payet, Farouk Dargai, Philippe Gasque, Xavier Guillot

**Affiliations:** 1 Research Unit ‘Etudes en Pharmaco-Immunologie’ UR EPI, Université de la Réunion, 97400 Réunion, France; philippe.gasque@gmail.com (P.G.); xavier.guillot@chu-reunion.fr (X.G.); 2 Orthopedic Clinical Department, CHU Bellepierre, Reunion University Hospital, 97400 Réunion, France; farouk.dargai@chu-reunion.fr; 3 Immunology Laboratory (LICE-OI), CHU Bellepierre, Reunion University Hospital, 97400 Réunion, France; 4 Rheumatology Clinical Department, CHU Bellepierre, Reunion University Hospital, 97400 Réunion, France

**Keywords:** rheumatoid arthritis epigenetic regulation, miR-155, miR-146a, miR-150, miR-410-3p, DNA methylation

## Abstract

The inflammatory reaction in rheumatoid arthritis (RA) is controlled by major epigenetic modifications that modulate the phenotype of synovial and immune cells. The aim of this work was to perform a systematic review focusing on miR expression, DNA methylation and histone modifications in RA. We demonstrated that, in human samples, the expressions of miR-155, miR-146a and miR-150 were significantly decreased while the expression of miR-410-3p was significantly increased in the RA group. Moreover, miR-146a significantly decreased pro-autoimmune IL-17 cytokine expression in RA. In a murine model, miR-34a inhibition can ameliorate the arthritis score. However, this evidence remain critically insufficient to support current therapeutic applications in RA patients.

## 1. Introduction

Rheumatoid arthritis (RA) is an auto-immune, chronic and inflammatory joint and systemic disease mediated by autoantibodies targeting immunoglobulin G (rheumatoid factor) and/or citrullinated proteins [1]: 50–80% of patients with RA have these rheumatoid factors and/or ACPAs (anti-citrullinated protein antibodies) [2]. RA is characterized by joint inflammation stimulated by inflammatory cell infiltrate (leading to synovitis) and structural damage. Moreover, RA is associated with a proliferation of activated synovial fibroblasts (SFs) [3].

In RA, inflammation is maintained by the overproduction of pro-inflammatory cytokines, such as TNF-α, known to play an important role in synovial inflammation and joint destruction [2]. TNF-α can induce elevated expressions of other pro-inflammatory cytokines such as IL-1, IL-6 and matrix metalloproteinases (MMPs), leading further to inflammation and joint destruction [4]. IL-1β also participates in inflammation by increasing the production of cytokines, particularly by SFs. Likewise, IL-17 increases the production of pro-inflammatory cytokines (TNFα, IL1β and IL-6) and MMPs [4].

In RA, synovial and cartilage cells are affected by joint inflammation. RA SFs have a major pathogenic phenotype and secrete pro-inflammatory cytokines such as, TNF-α, IL-1β, IL-6 and IL-17. In RA, SFs also secrete pro-angiogenic factors such as vascular endothelial factor (VEGF) and pro-osteoclastic factors (i.e., RANK-ligand (receptor of nuclear factor kappa-β ligand)). All of these agents promote the recruitment of pro-inflammatory cells in the synovial tissue and maintain chronic inflammation in the joints, therefore leading to tissue destruction, structural damage, and disability [5]. RA is also characterized by a systemic inflammation and causes an increased cardiovascular risk [1].

Macrophages such as synoviocytes [2], dendritic cells, and T and B cells also mediate inflammation in RA by secreting pro-inflammatory cytokines [6].

Human chondrocytes stimulated with IL-1β expressed TNF-β and TNF-β receptors. Moreover, TNF-β inhibited IL-1β-induced NF-kB activation [7].

The heritability of RA is 50–60%. Deane et al. described that genetic and environmental factors can participate in RA development [8]. Indeed, predisposition resulting in the generation of autoreactive T and B cells and several genetic factors including HLA-DR (Human Leukocyte Antigen- DR, gene polymorphisms called shared epitope), T cell and B cell function, and cytokine production are involved in RA development [6]. Environmental factors including viral and bacterial infections (such has *Porphyromonas gingivalis*) and other factors such as exposure to tobacco smoke, air pollution and obesity were described to increase RA risks. Conversely, moderate alcohol intake, healthy diet and statin use were described to decreased RA risk [8].

Epigenetic alterations including dysregulated micro-RNA (miRs) expression, DNA methylation and histone acetylation also play important roles in RA onset and pathogenesis by modulating the post-translational expression of key genes involved in synovial inflammation and joint destruction [1,5,9]. Epigenetic modifications can modulate gene activity and expression without changing DNA sequence [10].

**Figure 1:**
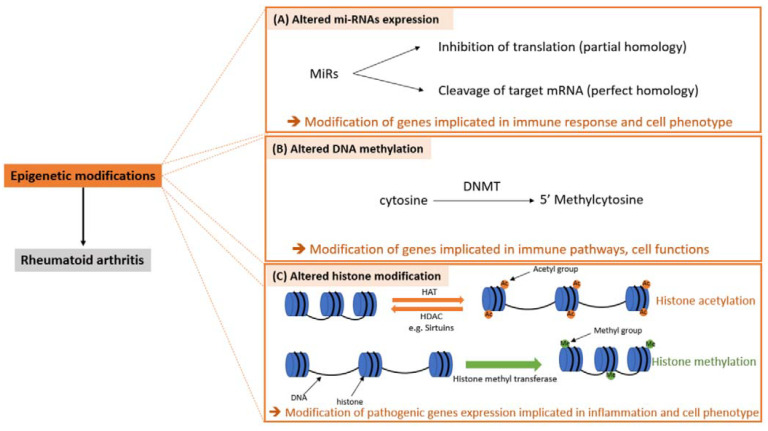
Epigenetic modifications in RA. DNMT: DNA methyl transferase, SF: synovial fibroblast, HAT: histone acetyl transferase, HDAC: histone deacetylase.

In RA, the aberrant expression of several miRs have been reported [11]. MiRs are small noncoding single-stranded-RNA that modulate gene expression through cleaving or inhibiting target mRNA [5,9] (Figure 1A). MiRs can modulate the expression of several genes involved in inflammation, cell apoptosis, cell proliferation, cartilage degradation and bone damage. MiRs are involved in macrophage, and B and T cell development and functions [12]. Moreover, in RA, miRs can modulate SF functions and can promote a switch toward a more pathogenic phenotype favoring the chronicity of the disease [5,9].

Aberrant DNA methylation has been reported in RA especially in immune peripheral blood cells [11]. DNA methylation is defined as an addition of the methyl group from S-adenosyl methionine to a cytosine base in CpG sites (DNA sites where the cytosine is followed by a guanine base). DNA methyl transferases (DNMT) catalyze the process of cytosine methylation [13] (Figure 1B). Methylation patterns can be obtained by two processes: de novo methylation or maintenance methylation [13]. In RA, there are global changes in DNA methylation. Moreover, in RA, promoters of different genes were hypomethylated or hypermethylated in different type of cells [14].

In RA, there is also histone modifications in immune cells. Histone modifications affect gene expression and determine the phenotype of the cells. Histone can be modified by different mechanisms including acetylation and methylation [11]. Histone acetylation is a reverse process. Indeed, histone acetyl transferases mediate the addition of an acetyl group (acetylation) and histone deacetylases (HDAC) mediate the removal of an acetyl group. In RA, the presence of pro-inflammatory cytokines in the joints plays an important role in histone acetylation. In RA, sirtuin (class III HDAC) expression is modified [15,16]. Sirtuins are NAD + HDAC involved in important metabolic pathways [17]. Sirtuins are involved in inflammation: sirtuin 1 [16] and sirtuin 6 [18] modulate the expression of genes involved in inflammation. Moreover, sirtuin 1 modulates the expression of genes involved in cell adhesion and modulates the proliferation of RA SF [16]. Sirtuins are also involved in osteoclast differentiation, and indeed, sirtuin 6 inhibits osteoclast differentiation [18] (Figure 1C).

The aim of this article was first to perform a systematic review of existing data about RA epigenetic regulation and then to list the current evidence concerning therapeutic applications and perspectives. In this meta-analysis, we showed that miR-155, miR-146a and miR-150 expressions were decreased in RA while miR-410-3p expression was increased compared with non-inflammatory controls. We also demonstrated that miR-146a decreased IL-17 protein expression in human cell culture. In murine model, we showed that the inhibition of miR-34a ameliorates the arthritis score. Furthermore, in RA, DNA seems to be hypomethylated.

## 2. Materials and Methods

The PRISMA statement checklist was used for meta-analysis and systematic review quality criteria [19].

### 2.1. Searching

We used the PubMed database. Search terms including micro-RNA and RA were used. The following keywords were used alone or combined: “rheumatoid arthritis + epigenetic regulation” (250 references), “rheumatoid arthritis + micro-RNA” (686 references), rheumatoid arthritis + DNA methylation” (268 references) and “rheumatoid arthritis + histone modifications” (95 references). We included only English language papers and original articles from 1976 to 2020. The screening was performed independently by two operators; then, a consensus was reached for study selection.

### 2.2. Eligibility Criteria and Study Selection

Studies focusing on the expression or pathophysiological effects of miRs in RA patients, human cell culture models or murine models of RA were selected. Review articles were excluded. We selected original articles. The quality of the studies was assessed (JADAD scale). Controlled studies were then selected. Among them, studies with quantified results related to differential miR expression levels and/or miR clinical, biological or structural effects were selected.

### 2.3. Statistical Analysis

We used the R device (meta and Rmeta libraries). Weighted mean differences (means ± SD) or pooled odds ratios were calculated. Intra- and then interclass effect sizes were assessed using funnel plots. The heterogeneity between studies was assessed (I^2^, tau-squared). The *p*-value was 5%. For meta-analysis, a fixed effect model or a random effect model was used depending on the heterogeneity between studies. Indeed, when there was heterogeneity among the studies, a random effect model was used, and a fixed model effect was used when there was no heterogeneity [20]. For meta-analysis different cell types were groupable due to the lack of studies on a specific cell type and the expression level scales were normalized in order to focus on intra- and interclass variations.

## 3. Results and Discussion

### 3.1. MiRs

#### 3.1.1. Study Selection and Characteristics

**Figure 2:**
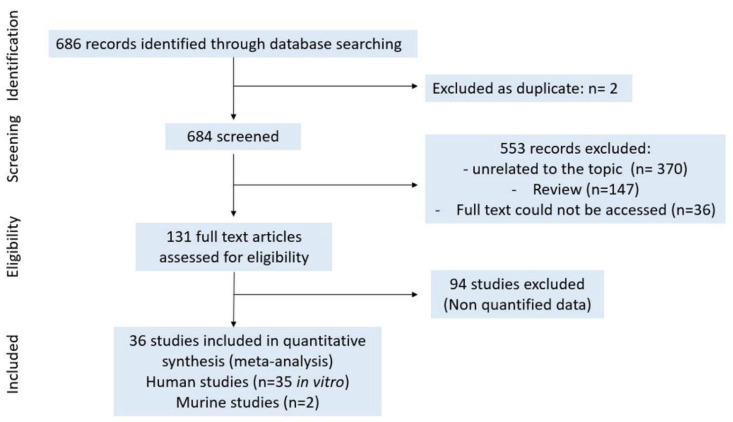
Study selection for miRs.

For miRs a, total of 686 results were found on PubMed on 9 October 2020 with the keywords “rheumatoid arthritis + micro-RNAs”. After removing duplicates (*n* = 2), 684 articles were screened. After title and abstract selection, 553 articles were excluded for the following reasons: unrelated to the topic (*n* = 370), review (*n* = 147) and full text not accessible (*n* = 36). In total, 131 full text articles were included. We included 36 studies in the quantitative analysis, and 94 studies were excluded because the data were not quantified (means/OR ± SD) and therefore could not be pooled (Figure 2).

#### 3.1.2. Quality Assessment

The quality of the included articles was assessed using the JADAD score with the following criteria: description of withdrawals and drop outs, objectives clearly defined, defined outcome measures, description of inclusion/exclusion criteria, sample size justified, clear description of methods, control group and description of statistical methods (Table 1).

#### 3.1.3. Modified Expression of miRs in RA: Meta-Analysis

The expressions of miR-16, miR-17, miR-22, miR-23b, miR-26a-5p, miR-124a, miR-125b, miR-132, miR-146a, miR-146b, miR-150, miR-155, miR-221, miR-223, miR-410-3p and miR-let-7a in patients with RA and in the control group were pooled and analyzed. Only four miRs showed significant results: miR-155, miR-146a, miR-150 and miR-410-3p.

**Figure 3:**
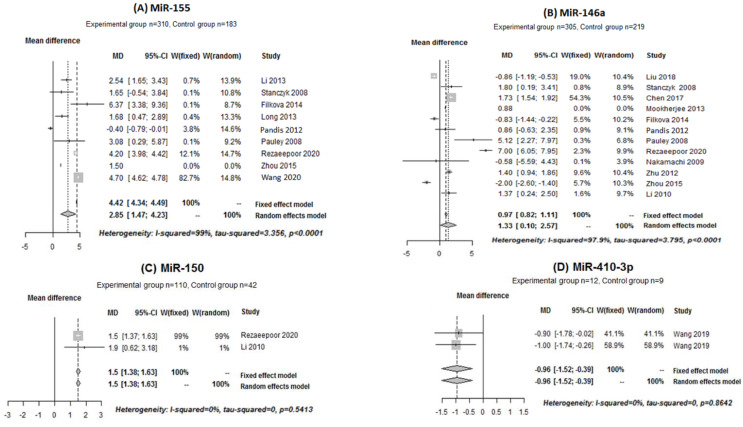
Forest plot of the meta-analysis for the expressions of (**A**–**D**) in human samples (PBMC, cell culture, sera and blood).

For miR-155 and miR-146a, there was heterogeneity among the included studies (I^2^ = 99%, tau-squared = 3.356 and *p* < 0.0001; I^2^ = 97.9%, tau-squared = 3.797 and *p* < 0.001, respectively). Thus, a random effect model was used for meta-analysis for these two miRs. However, there was no heterogeneity between the studies included for miR-150 (I^2^ = 0%, tau-squared = 0 and *p* = 0.5413) and miR-410-3p (I^2^ = 0%, tau-squared = 0 and *p* = 0.8642). Thus, a fixed effect model was used to pool the expression of these miRs. The mean differences of miR-155, miR-146, miR-150 and miR-410-3p versus controls were as follows (median (IC: 95%): 2.85 [1.47; 4.23], 1.33 [0.10; 2.57], 1.5 [1.38; 1.63] and −0.96 [−1.52; −0.39], respectively. These results indicated that miR-155 (Figure 3A), miR-146a (Figure 3B) and miR-150 (Figure 3C) were significantly increased in the RA patient group compared with the control patient group while miR-410-3p expression decreased (Figure 3D).

The pooled data for miR-16, miR-22 and miR-23b showed nonsignificant results (Supplementary Figure S1). After analyzing the pooled data, miR-26a-5p, miR-125b, miR-132, miR-146b, miR-221, miR-223 and miR-let-7a tended to show increased expressions in the RA group compared with the control group and miR-17 and miR-124a tended to show decreased expressions; however, the results were not significant (Supplementary Figures S2–S4).

#### 3.1.4. Effects of miRs on Pro-Inflammatory Cytokines Expression in Human Cell Culture: Meta-Analysis

**Figure 4:**
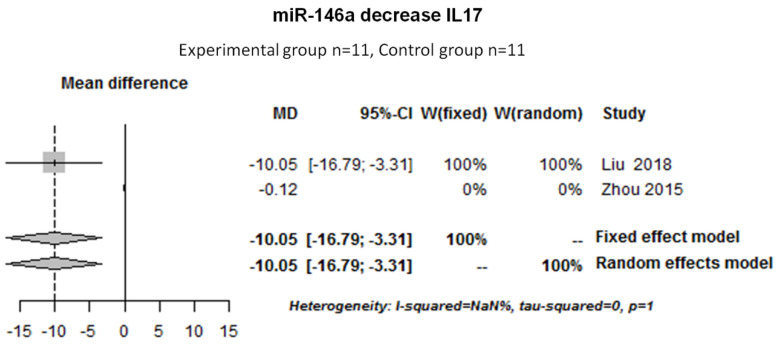
Forest plot of the meta-analysis for the effect of miR-146a on IL-17 in human cell cultures (SF, T cell). NaN: Not a number.

We also pooled and analyzed the data to determine the effect of miR-146a on cytokine expression. Two studies were pooled to determine the effect of miR-146a on IL-17. There was no heterogeneity among the studies for the effect of miR-146a on IL-17 (I^2^ = NaN%, tau-squared = 0 and *p* = 1). Thus, a fixed random effect model was used for the meta-analysis. The results showed that miR-146a decreased the IL-17 protein expression (−10.05 [−16.79; −3.31]) in human cell cultures (Figure 4).

Moreover, it also has been described that miR-146a decreased the expression of the TLR4/NF-kB pathway, leading a decrease in pro-inflammatory cytokines such as IL-1β, IL-6 and IL-8 [37].

We also pooled and analyzed the data for the effect of miR-155 on cytokines and MMPs. MiR-155 seemed to increase TNF-α, IL-1β and IL-6 protein expressions and decreased MMP-3 (metalloproteinase 3) mRNA expression. However, the results were not significant (Supplementary Figure S5).

It has been supposed that the increase in TNF-α and IL-1β expressions might be due to the decreased expression of SOCS1 (suppressor of cytokine signaling protein 1) by miR-155 [45]. Other studies demonstrated that miR-155 targets FOXO3a (forkhead box protein O3a) leading to the increased expression of TNF-α, IL-1β and IL-6 [48]. Moreover, other studies confirmed that miR-155 targeted FOXO3a as well as STAT1 [40], APAF-1 (apoptotic peptidase activating factor 1) and caspase 10 [47].

#### 3.1.5. Effects of miR-34a on Murine Arthritis Score: Meta-Analysis

**Figure 5:**
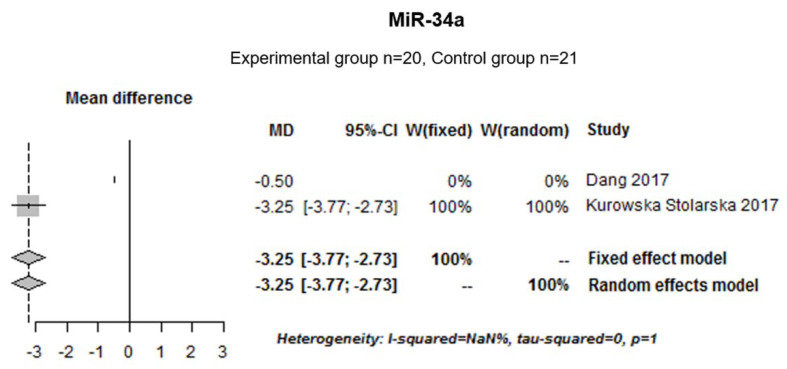
Forest plot meta-analysis for the effect of miR-34a inhibition on arthritis score (murine model).

Moreover, two animal studies performed with collagen-induced arthritis (experimental group *n* = 20; control group *n* = 21) were pooled to determine the effect of miR-34a on arthritis score. There was no heterogeneity among the studies (I^2^ = NaN%, tau-squared = 0 and *p* = 1). Thus, a fixed effect model was used. The median [IC 95%] was −3.25 [−3.77; −2.73] indicating that miR-34a inhibition significantly decrease arthritis score in murine models (Figure 5).

MiR-34a contributes to the development of RA by controlling Axl (AXL receptor tyrosine kinase), an inhibitor of dendritic cell auto-regulator [55]. The inhibition of miR-34a leads to a decrease in inflammatory-induced bone loss, Treg and pro-inflammatory cytokines [56].

### 3.2. DNA Methylation

#### 3.2.1. Study Selection and Characteristics

**Figure 6:**
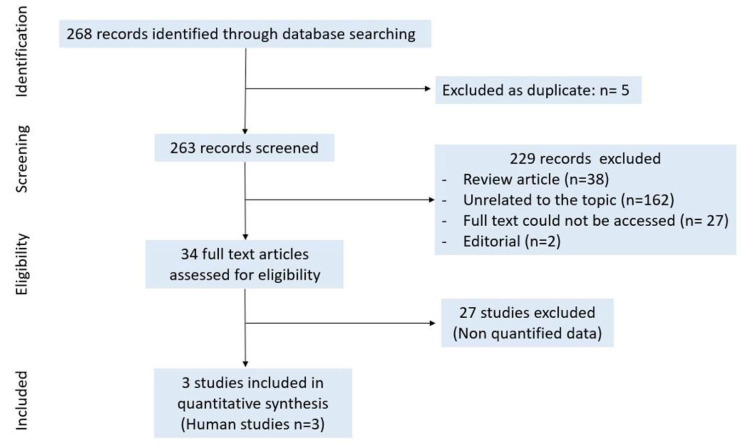
Study selection for DNA methylation.

The PubMed search displayed 268 records for “DNA methylation + rheumatoid arthritis” on 22 December 2020. In total, 5 duplicates were removed; 263 records were screened; and 229 records were excluded as review articles (*n* = 38), as not being related to the topic (*n* = 162), as full text could not be accessed (*n* = 27) or as editorials (*n* = 2). We included 34 full text articles after title and abstract selection. In the selected studies, three were included in the meta-analysis (Figure 6).

#### 3.2.2. Quality Assessment

As described below, the JADAD score was assessed to determine the quality of the studies (Table 2).

#### 3.2.3. DNA Methylation

**Figure 7:**
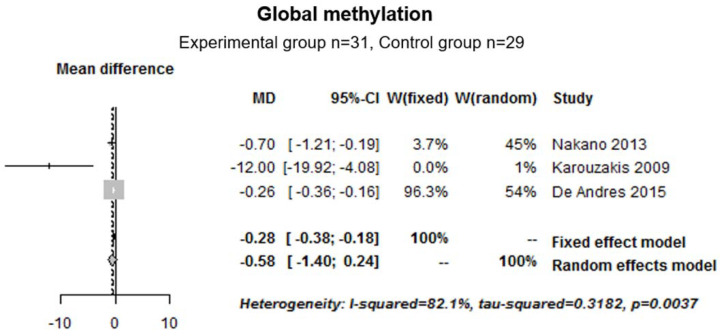
Forest plot for global methylation in human cell cultures.

First, we pooled and analyzed the global methylation data (human samples) from three studies (experimental group *n* = 31; control group *n* = 29). The median [IC 95%] was −0.58 [−1.40; 0.24], indicating that, in the RA group, DNA seemed to be hypomethylated compared with the control group; however, the results were not statistically significant. For this analysis, we used a random effect model to assess the meta-analysis. Indeed, there was heterogeneity between the studies (I^2^ = 82.1%, tau-squared = 0.3182 and *p* = 0.0037) (Figure 7).

It has been demonstrated that growth factors and receptors, extracellular matrix proteins, matrix-degrading enzymes and adhesion molecules are hypomethylated in RA [58]. More precisely, CHI3L1 (chitinase 3 like 1), caspase 1, STAT3 (signal transducer and activator of transcription 3), MAP3K5 (mitogen-activated protein kinase kinase kinase 5) and WISP3 (WNT1 Inducible signaling pathway protein 3 genes) were hypomethylated in RA [59].

**Figure 8:**
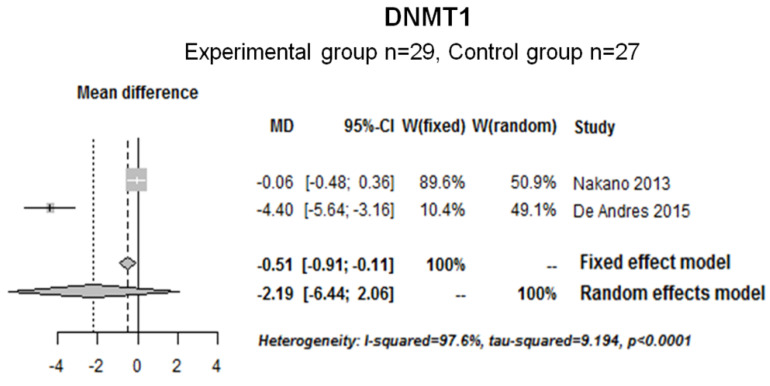
Forest plot for DNMT1 modified expression in human samples (T cell, SF).

Next, the DNMT1 (DNA methyl transferase 1) expression data from two studies (experimental group (*n* = 29); control group (*n* = 27)) were pooled and analyzed. Heterogeneity was observed between studies (I^2^ = 97.6%, tau-squared = 9.194 and *p* < 0.0001); thus, a random effect model was used. The results showed that DNMT1 seemed to be decreased in RA patients compared with the control group; however, the results were not significant (−2.19 [−6.44; 2.06]) (Figure 8).

### 3.3. Histone Modifications

#### Study Selection and Characteristics

**Figure 9:**
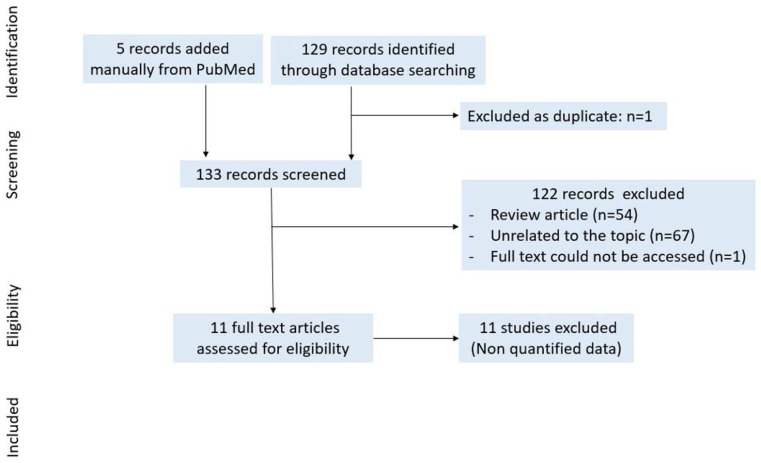
Study selection for histone modifications.

In total, 129 records were identified through the PubMed database searching for “histone modifications + rheumatoid arthritis” on 19 January 2021. Six articles were added manually from the PubMed search with “histone acetylation + rheumatoid arthritis”. Only 1 article was excluded as a duplicate, 133 records were screened, 122 records (review article (*n* = 54), unrelated to the topic (*n* = 67) and full text not accessible (*n* = 1)) were excluded after title and abstract selection, and 12 full texts were assessed for eligibility. We did not select studies for meta-analysis because data could not be pooled (Figure 9).

### 3.4. Discussion

In this systematic review of the literature, we first examined the specific expression of miRs in RA compared with non-inflammatory controls.

We showed that miR-155 was upregulated in RA patients. Moreover, we demonstrated that miR-155 tended to increase TNF-α, IL-1 and IL-6 protein expressions and tended to decrease MMP-3 mRNA expression compared with the control group in a human cell culture model. It has been demonstrated that miR-155 decreased the SOCS1 [45] and FOXO3a [48] expressions, leading to an increase in pro-inflammatory cytokines. Furthermore, miR-155 plays an important role in RA arthritis and was expressed by different type of cells. Indeed, miR-155 increased the inflammatory profile of CD14+ monocytes by increasing the expression of chemokines (such as MCP-1/CCL2) and pro-inflammatory cytokines (such as IL-6, IFN-α and TNF-α) [47,60]. MiR-155 also favored inflammation in RA SF [48]. In contrast, it has been described that miR-155 decreased the mRNA expression of APAF-1 and caspase 10 [47].

Next, we showed that, in RA, miR-146a was upregulated compared with the control group. We also demonstrated that miR-146a decreased IL-17 protein expression. Zhou et al. described that miR-146a increased STAT-1 (signal transducer and activator of transcription 1) activation, favoring a pro-inflammatory phenotype of Treg [43]. It has also been demonstrated that miR-146a decreased the expressions of TLR4 and NF-kB [37]. Moreover, further studies described the protective effect of miR-146a on bone erosion and osteoclastogenesis in murine models [37,61,62].

We also demonstrated that miR-150 was upregulated in RA patients while miR-410-3p was downregulated compared with the control group. Li et al. described that the expression of miR-150 was not correlated with the levels of pro-inflammatory cytokines [39]. It has also been speculated that miR-150 is an important molecule for T cell and B cell development. For instance, it has been demonstrated that the premature expression of miR-150 blocked the development of B cells [63].

Furthermore, miR-410-3p has been described to suppress proliferation while promoting apoptosis and G1-S phase transition through targeting YY1 (Yin Yang 1 transcription factor) in RA SF [52]. It has also been shown that, in RA, miR-410-3p suppressed pro-inflammatory cytokine expression by suppressing the NF-kB signaling pathway [53].

In murine models, we demonstrated that miR-34a inhibition significantly decreased the arthritis score. MiR-34a inhibition also decreased TNF-α, IL-1β, IL-6, IFN-γ, IL-17A and IL-21 transcripts in the synovium of collagen-induced arthritis mice injected with miR-34a antagomir [56]. Moreover, Hou et al. demonstrated that miR-34a-3p suppressed TNF-α and IL6 mRNA and protein expression in RA SF transfected with miR-34a-3p [64]. It has been shown that miR-34a targets Axl [55] and that this inhibition decreased bone loss, pro-inflammatory cytokine expression and Treg population [56]. These results suggest that miR-34a might play an important role in RA pathogenesis.

Concerning the therapeutic applications and perspectives of miRs, several studies used miR-atelocollagen complexes injected into the joints [65] or intravenously [62,66] in collagen-induced arthritis mice in order to overexpress specific miRs. The miR-atellocollagen complexes were chosen because these complexes are (1) resistant to nucleases, (2) efficiently transduced into cells and (3) prevented from diffusing out at the injected sites [65]. Currently, there are only in vitro studies concerning the role of miRs in RA in human models. Concerning the in vitro studies, miRs can be expressed in cell culture using different methods. The most current method is the use of transfection reagents such as lipofectamine [37,41,45,46], NTER transfection reagent [44,47,60] or oligofectamine [49]. Other studies used electroporation to deliver miRs into the cells [51,67,68]. Li et al. and Chen et al. performed miRs transfection using plasmid-encoded miRs [39,69]. Zhou et al. used serum-free ACCEL medium, which is an enriched serum media formulated to transfect siRNAs [43]. Another therapeutic application and perspective of miRs is the use of antagomirs. In several studies, antagomirs were transfected into human RA SF in vitro [31,46,64,70,71] to inhibit miR expression. Antagomirs were also used in murine models. For example, anti-miR-21 was transduced into rat cells using lentivirus encoding anti-miR-21 leading to the reduction of NF-kB protein levels [72]. Moreover, in in vivo murine models, antagomirs can be injected into mice [56,73].

Next, we examined the effect of DNA methylation on RA. We confirmed that DNA seems to be hypomethylated in the RA group and that DNMT1 levels seem to be decreased in RA. However, the data were not sufficient to perform quantified analysis. DNA methylation can alter gene expression; indeed, hypomethylation leads to increase gene expressions such as pro-inflammatory cytokine genes (e.g., IL-6 [74] and chemokines (e.g., CXCL12 (C-X-C motif chemokine ligand 12) [75]). It is important to note that, in RA, SF long-term exposure to IL-1 decreased DNMT1 mRNA expression [76]. One of the hypotheses is that DNA hypomethylation in RA might be due to DNMT1 deficiency. Indeed, several studies demonstrated that DNMT1 is associated with genomic hypomethylation [77,78,79]. Moreover, several genes were hypomethylated in RA such as CHI3L1 a cartilage-specific antigen, which is the target for autoimmunity in RA, caspase 1 and MAP3K5 implicated in apoptosis; STAT3, a key signaling protein activated in RA and associated with production of pro-inflammatory cytokines such as IL-6; and WISP3, which encodes growth factors [59]. Furthermore, it has been described that the CTL4-A (cytotoxic T-lymphocyte antigen 4), an inhibitory molecule regulating T cell function, promoter region in Treg was hypomethylated [80]. Furthermore, it has been shown that methotrexate treatment correlates with the decrease expression of DNMT1 and the reduced methylation of FOXP3, leading to the increased of FOXP3 mRNA expression in RA Treg [81]. DNA methylation can be related with miR expression. Indeed, in RA, SF miR-29 targets the expression of DNMT3A [82]. Nakano and Firestein showed that, in SF exposure to pro-inflammatory mediators such as IL-1β, TNF-α and LPS decreased the mRNA expressions of DNMT1 and DNMT3a [76].

Concerning therapeutic applications and perspectives of DNA methylation, the use of azacitidine as a treatment in vitro in human PBMC increased the IL-10 mRNA and protein expressions in RA [83]. Moreover, Neidhart et al. demonstrated that using diminazene aceturate, which is an inhibitor of SSAT-1 (spermidine/spermine N1-acetyltransferase 1), can restore the levels of DNA methylation and the expression of DNMT1 reducing RA SF invasive function [84].

In this study, we were unable to perform meta-analyzes on histone modifications given the limited data set and the heterogeneity among the studies. However, we can note that, in RA SF, there is an hyperacetylation of histone H3 in the IL-6 promoter leading to the increased production of IL-6 [85].

The use of HDAC inhibitors might be promising novel therapeutic avenues in RA. Several HDAC inhibitors have been described to ameliorate RA in vitro. The use of HDAC inhibitors such as ITF2357 suppressed the mRNA expression of IL-6, IL-8, CXCL12 and MMP-1 in RA SF stimulated with IL-1β [86]. Moreover, SAHA (suberoylanilide hydroxamic acid), another inhibitor of HDAC, inhibited viability and induced apoptotic death in RA SF [87]. Treatment with romidepsin (FK228) induced cell arrest after stimulation with TNF-α and IL-1β [88]. HDAC inhibitors have also been studied on the murine model. In CIA mice, valproic acid decreased the histopathologic score associated with CIA and increased Treg functions, leading to the improvement of clinical disease [89].

In this systematic review, we focused on the aberrant expression of miRs, DNA methylation and histone modifications on RA. However, it is important to note that phytopharmaceuticals natural products such as curcumin [90] and resveratrol [91,92], nutrition and environmental conditions can influence chronic diseases such as RA.

In this meta-analysis, some limitations should be considered as follows: (1) there was not enough groupable data for a specific cell type. We had to group all of the cell types to obtain analyzable data; (2) cell models were very varied; (3) there was heterogeneity for some of the meta-analyzes; (4) for some studies, there was small sample sizes; and (5) we included only studies published in English.

## 4. Conclusions

In summary, this is the first meta-analysis demonstrating that miR-155, miR-146 and miR-150 expressions increased and miR-410-3p expression decreased in human RA cell culture models compared with the control groups. We also demonstrated that miR-146a decreased the IL-17 protein expression in human cell culture model. Moreover, in a murine model, miR-34a inhibition can ameliorate the arthritis score. However, these results remain to be confirmed in human studies. Then, the injection of miR-atellocollagen complexes might be a promising method to deliver miRs at the targeted site. Antagomirs might be a promising method to inhibit miRs and to ameliorate RA.

**Figure 10:**
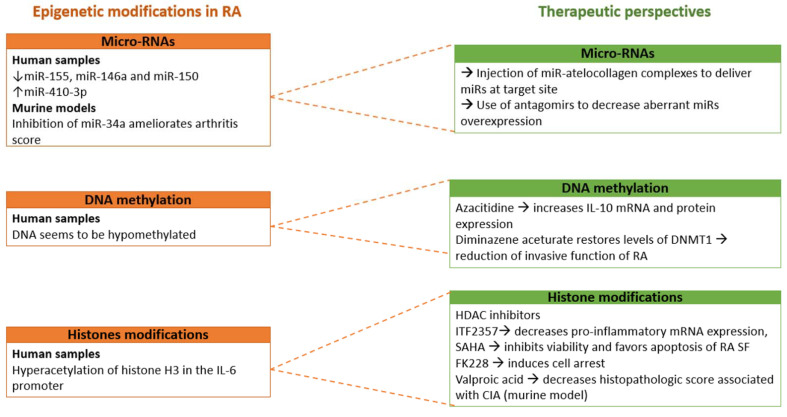
Conclusion scheme.

We also confirmed that DNA seems to be hypomethylated in RA. Moreover, the use of DNMT inhibitors such as azacitidine and diminazene aceturate, an inhibitor of SSAT-1, could be promising therapeutic avenues to ameliorate RA. Finally, inhibitors of HDAC might be a promising future therapeutic target in RA (Figure 10).

Concerning the therapeutic application in humans, currently, the levels of evidence remain insufficient. Further studies are required in order to better characterize the influence of epigenetic modifications in RA patients.

## Figures and Tables

**Table 1:** Characteristics of the studies included in mi-RNAs meta-analysis. PBMC: peripheral blood mononuclear cell, ST: synovial tissue, CIA: collagen-induced arthritis.

References	MiRs	Samples	JADAD Score/8
Wei et al., 2020 [21]	miR-16	SF	6
Pauley et al., 2008 [22]	miR-16	PBMC	6
Filkova et al., 2014 [23]	miR-16	Sera	6
Akhtar et al., 2016 [24]	miR-17	SF	6
Wang et al., 2018 [25]	miR-17	T reg	6
Lin et al., 2014 [26]	miR-22	ST and SF	6
Zhang et al., 2020 [27]	miR-22	SF	6
Liu et al., 2019 [28]	miR-23b	ST	6
Zhu et al., 2012 [29]	miR-23b	SF	6
Dunaeva et al., 2018 [30]	miR-26a-5p	Sera	6
Huang et al., 2019 [31]	miR-26a-5p	SF	6
Li et al., 2018 [32]	miR-124a	SF	6
Nakamachi et al., 2009 [33]	miR-124a	SF	6
Duroux-Richard et al., 2014 [34]	miR-125b	Blood	6
Cheng and Wang, 2020 [35]	miR-125b	Blood	6
Pauley et al., 2008 [22]	miR-132	PBMC	6
Filkova et al., 2014 [23]	miR-132	sera	6
Nakamachi et al., 2009 [33]	miR-146a	SF	6
Chen et al., 2017 [36]	miR-146a	PBMC	6
Liu et al., 2018 [37]	miR-146a	Tissue, SF	6
Mookherjee and El-Gabalawy, 2013 [38]	miR-146a	PBMC	6
Li et al., 2010 [39]	miR-146a, miR-146b	CD14+ cells	6
Rezaeepoor et al., 2020 [40]	miR-146a, miR-146b, miR-155	PBMC	6
Stanczyk et al., 2008 [41]	miR-146a, miR-155	SF	6
Pandis et al., 2012 [42]	miR-146a, miR-155	SF	6
Zhou et al., 2015 [43]	miR-146a, miR-155	T cell	6
Pauley et al., 2008 [22]	miR-146a, miR-155	PBMC	6
Filkova et al., 2014 [23]	miR-146a, miR-155	sera	6
Kurowska-Stolarska et al., 2011 [44]	miR-155	biopsies	5
Li et al., 2013 [45]	miR-155	PBMC	6
Long et al., 2013 [46]	miR-155	SF	6
Rajasekhar et al., 2017 [47]	miR-155	Synovial fluid	6
Wang et al., 2020 [48]	miR-155	ST	6
Li et al., 2010 [39]	miR-150	CD14+ cells	6
Rezaeepoor et al., 2020 [40]	miR-150	PBMC	6
Yang and Yang, 2015 [49]	miR-221	SF	6
Filkova et al., 2014 [23]	miR-221, miR-223	sera	6
Li et al., 2012 [50]	miR-223	Bone marrow macrophage	6
Lu et al., 2014 [51]	miR-223	T cell	6
Rezaeepoor et al., 2020 [40]	miR-223	PBMC	6
Wang et al., 2019 [52]	miR-410-3p	SF	6
Wang et al., 2019 [53]	miR-410-3p	SF	5
Zhu et al., 2017 [54]	miR-let-7a	Macrophage	6
Pauley et al., 2008 [22]	miR-let-7a	PBMC	6
Kurowska-Stolarska et al., 2017 [55]	miR-34a	CIA mice	5
Dang et al., 2017 [56]	miR-34a	CIA mice	5

**Table 2:** Characteristics of the studies included in the DNA methylation meta-analysis.

References	DNA Methylation in RA	Model	JADAD Score/8
De Andres et al., 2015 [57]	Global DNA methylation		6
	DNMT1	T cell, B cell, monocyte	
Karouazaki et al., 2009 [58]	Global DNA methylation; DNMT1	ST/SF	6
Nakano et al., 2013 [59]	Global DNA methylation	SF	6

## Data Availability

On demand—EPI research unit.

## Supplementary Materials

The following are available online at https://www.mdpi.com/article/10.3390/ijms222212170/s1, Figure S1: Forest plot of the meta-analysis for the expression of miR-16, miR-22 and miR-23b in human samples, Figure S2: Forest plot of the meta-analysis for the expressions of miR-26a-5p, miR-125b, miR-132, miR-146b, miR-221, miR-223 and miR-let-7a in human samples, Figure S3: Forest plot of the meta-analysis for the expressions of miR-17 and miR-124a in human samples, Figure S4: Forest plot of the meta-analysis for the effect of miR-155 on IL-6 and MMP-3 in human samples, Figure S5: Forest plot of the meta-analysis for the effect of miR-155 on IL-6 and MMP-3 in human samples.

## Abbreviations

APAF-1	Apoptotic Peptidase Activating Factor 1
Axl	AXL receptor tyrosine kinase
CHI3L1	Chitinase 3 Like 1
CIA	Collagen-Induced Arthritis
CTLA-4	Cytotoxic T-Lymphocyte Antigen 4
CXCL12	C-X-C Motif Chemokine Ligand 12 (also known as SDF1 alpha)
DNMT	DNA Methyl Transferase
FK228	romidepsin
FOXO3a	Forkhead box Protein O3a
HLA-DR	Human Leucocyte Antigen-DR
HDAC	Histone Deacetylase
IL	Interleukin
MAP3K5	Mitogen-Activated Protein Kinase Kinase Kinase 5
MiRs	Micro-RNAs
MMP	Metalloproteinase
NaN%	Not a number
PBMC	Peripheral Blood Mononuclear Cell
RA	Rheumatoid Arthritis
RANK-L	Receptor of Nuclear factor Kappa- β Ligand
SF	Synovial Fibroblasts
SOCS1	suppressor of cytokine signaling protein 1
SSAT-1	Spermidine/Spermine N1-AcetylTransferase 1
ST	Synovial Tissue
STAT-1	Signal Transducer and Activator of Transcription 1
STAT3	Signal Transducer and Activator of Transcription 3
TNF	Tumor Necrosis Alfa
VEGF	Vascular Endothelial Growth Factor
WISP3	WNT1 Inducible Signaling pathway Protein 3
YY1	Yin Yang 1 transcription factor

## References

[1] Smolen J.S., Aletaha D., Barton A., Burmester G.R., Emery P., Firestein G.S., Kavanaugh A., McInnes I.B., Solomon D.H., Strand V. (2018). Rheumatoid Arthritis. Nat. Rev. Dis. Primers.

[2] Scott D.L., Wolfe F., Huizinga T.W. (2010). Rheumatoid Arthritis. Lancet.

[3] Wasserman A.M. (2011). Diagnosis and Management of Rheumatoid Arthritis. Am. Fam. Physician.

[4] Mateen S., Zafar A., Moin S., Khan A.Q., Zubair S. (2016). Understanding the Role of Cytokines in the Pathogenesis of Rheumatoid Arthritis. Clin. Chim. Acta.

[5] Bottini N., Firestein G.S. (2013). Duality of Fibroblast-like Synoviocytes in RA: Passive Responders and Imprinted Aggressors. Nat. Rev. Rheumatol..

[6] Lin Y.-J., Anzaghe M., Schülke S. (2020). Update on the Pathomechanism, Diagnosis, and Treatment Options for Rheumatoid Arthritis. Cells.

[7] Buhrmann C., Shayan P., Aggarwal B.B., Shakibaei M. (2013). Evidence That TNF-β (Lymphotoxin α) Can Activate the Inflammatory Environment in Human Chondrocytes. Arthritis Res. Ther..

[8] Deane K.D., Demoruelle M.K., Kelmenson L.B., Kuhn K.A., Norris J.M., Holers V.M. (2017). Genetic and Environmental Risk Factors for Rheumatoid Arthritis. Best Pract. Res. Clin. Rheumatol..

[9] Nygaard G., Firestein G.S. (2020). Restoring Synovial Homeostasis in Rheumatoid Arthritis by Targeting Fibroblast-like Synoviocytes. Nat. Rev. Rheumatol..

[10] Doody K.M., Bottini N., Firestein G.S. (2017). Epigenetic Alterations in Rheumatoid Arthritis Fibroblast-like Synoviocytes. Epigenomics.

[11] Nemtsova M.V., Zaletaev D.V., Bure I.V., Mikhaylenko D.S., Kuznetsova E.B., Alekseeva E.A., Beloukhova M.I., Deviatkin A.A., Lukashev A.N., Zamyatnin A.A. (2019). Epigenetic Changes in the Pathogenesis of Rheumatoid Arthritis. Front. Genet..

[12] Ibáñez-Cabellos J.S., Seco-Cervera M., Osca-Verdegal R., Pallardó F.V., García-Giménez J.L. (2019). Epigenetic Regulation in the Pathogenesis of Sjögren Syndrome and Rheumatoid Arthritis. Front. Genet..

[13] Cribbs A., Feldmann M., Oppermann U. (2015). Towards an Understanding of the Role of DNA Methylation in Rheumatoid Arthritis: Therapeutic and Diagnostic Implications. Ther. Adv. Musculoskelet. Dis..

[14] Klein K., Gay S. (2015). Epigenetics in Rheumatoid Arthritis. Curr. Opin. Rheumatol..

[15] Kara M., Yolbaş S., Şahin C., Koca S.S. (2017). Changes in Sirtuin 2 and Sirtuin 3 MRNA Expressions in Rheumatoid Arthritis. Eur. J. Rheumatol..

[16] Engler A., Tange C., Frank-Bertoncelj M., Gay R.E., Gay S., Ospelt C. (2016). Regulation and Function of SIRT1 in Rheumatoid Arthritis Synovial Fibroblasts. J. Mol. Med..

[17] Sosnowska B., Mazidi M., Penson P., Gluba-Brzózka A., Rysz J., Banach M. (2017). The Sirtuin Family Members SIRT1, SIRT3 and SIRT6: Their Role in Vascular Biology and Atherogenesis. Atherosclerosis.

[18] Lee H.-S., Ka S.-O., Lee S.-M., Lee S.-I., Park J.-W., Park B.-H. (2013). Overexpression of Sirtuin 6 Suppresses Inflammatory Responses and Bone Destruction in Mice with Collagen-Induced Arthritis. Arthritis Rheum..

[19] Moher D., Shamseer L., Clarke M., Ghersi D., Liberati A., Petticrew M., Shekelle P., Stewart L.A., PRISMA-P Group (2015). Preferred Reporting Items for Systematic Review and Meta-Analysis Protocols (PRISMA-P) 2015 Statement. Syst. Rev..

[20] Barili F., Parolari A., Kappetein P.A., Freemantle N. (2018). Statistical Primer: Heterogeneity, Random- or Fixed-Effects Model Analyses?. Interact. Cardiovasc. Thorac. Surg..

[21] Wei H., Wu Q., Shi Y., Luo A., Lin S., Feng X., Jiang J., Zhang M., Wang F., Tan W. (2020). MicroRNA-15a/16/SOX5 Axis Promotes Migration, Invasion and Inflammatory Response in Rheumatoid Arthritis Fibroblast-like Synoviocytes. Aging.

[22] Pauley K.M., Satoh M., Chan A.L., Bubb M.R., Reeves W.H., Chan E.K. (2008). Upregulated MiR-146a Expression in Peripheral Blood Mononuclear Cells from Rheumatoid Arthritis Patients. Arthritis Res. Ther..

[23] Filková M., Aradi B., Senolt L., Ospelt C., Vettori S., Mann H., Filer A., Raza K., Buckley C.D., Snow M. (2014). Association of Circulating MiR-223 and MiR-16 with Disease Activity in Patients with Early Rheumatoid Arthritis. Ann. Rheum. Dis..

[24] Akhtar N., Singh A.K., Ahmed S. (2016). MicroRNA-17 Suppresses TNF-α Signaling by Interfering with TRAF2 and CIAP2 Association in Rheumatoid Arthritis Synovial Fibroblasts. J. Immunol..

[25] Wang L., Wang C., Jia X., Yu J. (2018). Circulating Exosomal MiR-17 Inhibits the Induction of Regulatory T Cells via Suppressing TGFBR II Expression in Rheumatoid Arthritis. Cell Physiol. Biochem..

[26] Lin J., Huo R., Xiao L., Zhu X., Xie J., Sun S., He Y., Zhang J., Sun Y., Zhou Z. (2014). A Novel P53/MicroRNA-22/Cyr61 Axis in Synovial Cells Regulates Inflammation in Rheumatoid Arthritis. Arthritis Rheumatol..

[27] Zhang C., Fang L., Liu X., Nie T., Li R., Cui L., Wang J., Ji Y. (2020). MiR-22 Inhibits Synovial Fibroblasts Proliferation and Proinflammatory Cytokine Production in RASF via Targeting SIRT1. Gene.

[28] Liu X., Ni S., Li C., Xu N., Chen W., Wu M., van Wijnen A.J., Wang Y. (2019). Circulating MicroRNA-23b as a New Biomarker for Rheumatoid Arthritis. Gene.

[29] Zhu S., Pan W., Song X., Liu Y., Shao X., Tang Y., Liang D., He D., Wang H., Liu W. (2012). The MicroRNA MiR-23b Suppresses IL-17-Associated Autoimmune Inflammation by Targeting TAB2, TAB3 and IKK-α. Nat. Med..

[30] Dunaeva M., Blom J., Thurlings R., Pruijn G.J.M. (2018). Circulating Serum MiR-223-3p and MiR-16-5p as Possible Biomarkers of Early Rheumatoid Arthritis. Clin. Exp. Immunol..

[31] Huang Z., Xing S., Liu M., Deng W., Wang Y., Huang Z., Huang Y., Huang X., Wu C., Guo X. (2019). MiR-26a-5p Enhances Cells Proliferation, Invasion, and Apoptosis Resistance of Fibroblast-like Synoviocytes in Rheumatoid Arthritis by Regulating PTEN/PI3K/AKT Pathway. Biosci. Rep..

[32] Li J., Song Q., Shao L., Zhang L.-L., Guo X.-H., Mao Y.-J. (2018). MiR-124a Inhibits Proliferation and Invasion of Rheumatoid Arthritis Synovial Fibroblasts. Eur. Rev. Med. Pharmacol. Sci..

[33] Nakamachi Y., Kawano S., Takenokuchi M., Nishimura K., Sakai Y., Chin T., Saura R., Kurosaka M., Kumagai S. (2009). MicroRNA-124a Is a Key Regulator of Proliferation and Monocyte Chemoattractant Protein 1 Secretion in Fibroblast-like Synoviocytes from Patients with Rheumatoid Arthritis. Arthritis Rheum..

[34] Duroux-Richard I., Pers Y.-M., Fabre S., Ammari M., Baeten D., Cartron G., Touitou I., Jorgensen C., Apparailly F. (2014). Circulating MiRNA-125b Is a Potential Biomarker Predicting Response to Rituximab in Rheumatoid Arthritis. Mediat. Inflamm..

[35] Cheng P., Wang J. (2020). The Potential of Circulating MicroRNA-125a and MicroRNA-125b as Markers for Inflammation and Clinical Response to Infliximab in Rheumatoid Arthritis Patients. J. Clin. Lab. Anal..

[36] Chen Z.-Z., Zhang X.-D., Chen Y., Wu Y.-B. (2017). The Role of Circulating MiR-146a in Patients with Rheumatoid Arthritis Treated by Tripterygium Wilfordii Hook F. Medicine.

[37] Liu W., Wu Y.-H., Zhang L., Xue B., Wang Y., Liu B., Liu X.-Y., Zuo F., Yang X.-Y., Chen F.-Y. (2018). MicroRNA-146a Suppresses Rheumatoid Arthritis Fibroblast-like Synoviocytes Proliferation and Inflammatory Responses by Inhibiting the TLR4/NF-KB Signaling. Oncotarget.

[38] Mookherjee N., El-Gabalawy H.S. (2013). High Degree of Correlation between Whole Blood and PBMC Expression Levels of MiR-155 and MiR-146a in Healthy Controls and Rheumatoid Arthritis Patients. J. Immunol. Methods.

[39] Li J., Wan Y., Guo Q., Zou L., Zhang J., Fang Y., Zhang J., Zhang J., Fu X., Liu H. (2010). Altered MicroRNA Expression Profile with MiR-146a Upregulation in CD4+ T Cells from Patients with Rheumatoid Arthritis. Arthritis Res. Ther..

[40] Rezaeepoor M., Pourjafar M., Tahamoli-Roudsari A., Basiri Z., Hajilooi M., Solgi G. (2020). Altered Expression of MicroRNAs May Predict Therapeutic Response in Rheumatoid Arthritis Patients. Int. Immunopharmacol..

[41] Stanczyk J., Pedrioli D.M.L., Brentano F., Sanchez-Pernaute O., Kolling C., Gay R.E., Detmar M., Gay S., Kyburz D. (2008). Altered Expression of MicroRNA in Synovial Fibroblasts and Synovial Tissue in Rheumatoid Arthritis. Arthritis Rheum..

[42] Pandis I., Ospelt C., Karagianni N., Denis M.C., Reczko M., Camps C., Hatzigeorgiou A.G., Ragoussis J., Gay S., Kollias G. (2012). Identification of MicroRNA-221/222 and MicroRNA-323-3p Association with Rheumatoid Arthritis via Predictions Using the Human Tumour Necrosis Factor Transgenic Mouse Model. Ann. Rheum. Dis..

[43] Zhou Q., Haupt S., Kreuzer J.T., Hammitzsch A., Proft F., Neumann C., Leipe J., Witt M., Schulze-Koops H., Skapenko A. (2015). Decreased Expression of MiR-146a and MiR-155 Contributes to an Abnormal Treg Phenotype in Patients with Rheumatoid Arthritis. Ann. Rheum. Dis..

[44] Kurowska-Stolarska M., Alivernini S., Ballantine L.E., Asquith D.L., Millar N.L., Gilchrist D.S., Reilly J., Ierna M., Fraser A.R., Stolarski B. (2011). MicroRNA-155 as a Proinflammatory Regulator in Clinical and Experimental Arthritis. Proc. Natl. Acad. Sci. USA.

[45] Li X., Tian F., Wang F. (2013). Rheumatoid Arthritis-Associated MicroRNA-155 Targets SOCS1 and Upregulates TNF-α and IL-1β in PBMCs. Int. J. Mol. Sci..

[46] Long L., Yu P., Liu Y., Wang S., Li R., Shi J., Zhang X., Li Y., Sun X., Zhou B. (2013). Upregulated MicroRNA-155 Expression in Peripheral Blood Mononuclear Cells and Fibroblast-like Synoviocytes in Rheumatoid Arthritis. Clin. Dev. Immunol..

[47] Rajasekhar M., Olsson A.M., Steel K.J.A., Georgouli M., Ranasinghe U., Brender Read C., Frederiksen K.S., Taams L.S. (2017). MicroRNA-155 Contributes to Enhanced Resistance to Apoptosis in Monocytes from Patients with Rheumatoid Arthritis. J. Autoimmun..

[48] Wang Y., Feng T., Duan S., Shi Y., Li S., Zhang X., Zhang L. (2020). MiR-155 Promotes Fibroblast-like Synoviocyte Proliferation and Inflammatory Cytokine Secretion in Rheumatoid Arthritis by Targeting FOXO3a. Exp. Ther. Med..

[49] Yang S., Yang Y. (2015). Downregulation of MicroRNA-221 Decreases Migration and Invasion in Fibroblast-like Synoviocytes in Rheumatoid Arthritis. Mol. Med. Rep..

[50] Li Y.-T., Chen S.-Y., Wang C.-R., Liu M.-F., Lin C.-C., Jou I.-M., Shiau A.-L., Wu C.-L. (2012). Brief Report: Amelioration of Collagen-Induced Arthritis in Mice by Lentivirus-Mediated Silencing of MicroRNA-223. Arthritis Rheum..

[51] Lu M.-C., Yu C.-L., Chen H.-C., Yu H.-C., Huang H.-B., Lai N.-S. (2014). Increased MiR-223 Expression in T Cells from Patients with Rheumatoid Arthritis Leads to Decreased Insulin-like Growth Factor-1-Mediated Interleukin-10 Production. Clin. Exp. Immunol..

[52] Wang Y., Jiao T., Fu W., Zhao S., Yang L., Xu N., Zhang N. (2019). MiR-410-3p Regulates Proliferation and Apoptosis of Fibroblast-like Synoviocytes by Targeting YY1 in Rheumatoid Arthritis. Biomed. Pharmacother..

[53] Wang Y., Xu N., Zhao S., Jiao T., Fu W., Yang L., Zhang N. (2019). MiR-410-3p Suppresses Cytokine Release from Fibroblast-Like Synoviocytes by Regulating NF-ΚB Signaling in Rheumatoid Arthritis. Inflammation.

[54] Zhu W., Yu J., Qiu S., Liu H., Wang Y., Xu X., Shao L., Zhu L., Jiao Y., Liu F. (2017). MiR-Let-7a Regulates Anti-Citrullinated Protein Antibody-Induced Macrophage Activation and Correlates with the Development of Experimental Rheumatoid Arthritis. Int. Immunopharmacol..

[55] Kurowska-Stolarska M., Alivernini S., Melchor E.G., Elmesmari A., Tolusso B., Tange C., Petricca L., Gilchrist D.S., Di Sante G., Keijzer C. (2017). MicroRNA-34a Dependent Regulation of AXL Controls the Activation of Dendritic Cells in Inflammatory Arthritis. Nat. Commun..

[56] Dang Q., Yang F., Lei H., Liu X., Yan M., Huang H., Fan X., Li Y. (2017). Inhibition of MicroRNA-34a Ameliorates Murine Collagen-Induced Arthritis. Exp. Ther. Med..

[57] de Andres M.C., Perez-Pampin E., Calaza M., Santaclara F.J., Ortea I., Gomez-Reino J.J., Gonzalez A. (2015). Assessment of Global DNA Methylation in Peripheral Blood Cell Subpopulations of Early Rheumatoid Arthritis before and after Methotrexate. Arthritis Res. Ther..

[58] Karouzakis E., Gay R.E., Michel B.A., Gay S., Neidhart M. (2009). DNA Hypomethylation in Rheumatoid Arthritis Synovial Fibroblasts. Arthritis Rheum..

[59] Nakano K., Whitaker J.W., Boyle D.L., Wang W., Firestein G.S. (2013). DNA Methylome Signature in Rheumatoid Arthritis. Ann. Rheum. Dis..

[60] Elmesmari A., Fraser A.R., Wood C., Gilchrist D., Vaughan D., Stewart L., McSharry C., McInnes I.B., Kurowska-Stolarska M. (2016). MicroRNA-155 Regulates Monocyte Chemokine and Chemokine Receptor Expression in Rheumatoid Arthritis. Rheumatology.

[61] Ammari M., Presumey J., Ponsolles C., Roussignol G., Roubert C., Escriou V., Toupet K., Mausset-Bonnefont A.-L., Cren M., Robin M. (2018). Delivery of MiR-146a to Ly6Chigh Monocytes Inhibits Pathogenic Bone Erosion in Inflammatory Arthritis. Theranostics.

[62] Nakasa T., Shibuya H., Nagata Y., Niimoto T., Ochi M. (2011). The Inhibitory Effect of MicroRNA-146a Expression on Bone Destruction in Collagen-Induced Arthritis. Arthritis Rheum..

[63] Zhou B., Wang S., Mayr C., Bartel D.P., Lodish H.F. (2007). MiR-150, a MicroRNA Expressed in Mature B and T Cells, Blocks Early B Cell Development When Expressed Prematurely. Proc. Natl. Acad. Sci. USA.

[64] Hou C., Wang D., Zhang L. (2019). MicroRNA-34a-3p Inhibits Proliferation of Rheumatoid Arthritis Fibroblast-like Synoviocytes. Mol. Med. Rep..

[65] Lee W.S., Yasuda S., Kono M., Kudo Y., Shimamura S., Kono M., Fujieda Y., Kato M., Oku K., Shimizu T. (2020). MicroRNA-9 Ameliorates Destructive Arthritis through down-Regulation of NF-ΚB1-RANKL Pathway in Fibroblast-like Synoviocytes. Clin. Immunol..

[66] Murata K., Furu M., Yoshitomi H., Ishikawa M., Shibuya H., Hashimoto M., Imura Y., Fujii T., Ito H., Mimori T. (2013). Comprehensive MicroRNA Analysis Identifies MiR-24 and MiR-125a-5p as Plasma Biomarkers for Rheumatoid Arthritis. PLoS ONE.

[67] Gao J., Zhou X.-L., Kong R.-N., Ji L.-M., He L.-L., Zhao D.-B. (2016). MicroRNA-126 Targeting PIK3R2 Promotes Rheumatoid Arthritis Synovial Fibro-Blasts Proliferation and Resistance to Apoptosis by Regulating PI3K/AKT Pathway. Exp. Mol. Pathol..

[68] Philippe L., Alsaleh G., Suffert G., Meyer A., Georgel P., Sibilia J., Wachsmann D., Pfeffer S. (2012). TLR2 Expression Is Regulated by MicroRNA MiR-19 in Rheumatoid Fibroblast-like Synoviocytes. J. Immunol..

[69] Chen Z., Wang H., Xia Y., Yan F., Lu Y. (2018). Therapeutic Potential of Mesenchymal Cell-Derived MiRNA-150-5p-Expressing Exosomes in Rheumatoid Arthritis Mediated by the Modulation of MMP14 and VEGF. J. Immunol..

[70] Sun J., Yan P., Chen Y., Chen Y., Yang J., Xu G., Mao H., Qiu Y. (2015). MicroRNA-26b Inhibits Cell Proliferation and Cytokine Secretion in Human RASF Cells via the Wnt/GSK-3β/β-Catenin Pathway. Diagn. Pathol..

[71] Wang X., Tang K., Wang Y., Chen Y., Yang M., Gu C., Wang J., Wang Y., Yuan Y. (2019). Elevated MicroRNA-145-5p Increases Matrix Metalloproteinase-9 by Activating the Nuclear Factor-κB Pathway in Rheumatoid Arthritis. Mol. Med. Rep..

[72] Chen Y., Xian P.-F., Yang L., Wang S.-X. (2016). MicroRNA-21 Promotes Proliferation of Fibroblast-Like Synoviocytes through Mediation of NF-ΚB Nuclear Translocation in a Rat Model of Collagen-Induced Rheumatoid Arthritis. Biomed. Res. Int..

[73] Tao Y., Wang Z., Wang L., Shi J., Guo X., Zhou W., Wu X., Liu Y., Zhang W., Yang H. (2017). Downregulation of MiR-106b Attenuates Inflammatory Responses and Joint. Damage in Collagen-Induced Arthritis. Rheumatology.

[74] Nile C.J., Read R.C., Akil M., Duff G.W., Wilson A.G. (2008). Methylation Status of a Single CpG Site in the IL6 Promoter Is Related to IL6 Messenger RNA Levels and Rheumatoid Arthritis. Arthritis Rheum..

[75] Karouzakis E., Rengel Y., Jüngel A., Kolling C., Gay R.E., Michel B.A., Tak P.P., Gay S., Neidhart M., Ospelt C. (2011). DNA Methylation Regulates the Expression of CXCL12 in Rheumatoid Arthritis Synovial Fibroblasts. Genes Immun..

[76] Nakano K., Boyle D.L., Firestein G.S. (2013). Regulation of DNA Methylation in Rheumatoid Arthritis Synoviocytes. J. Immunol..

[77] Turek-Plewa J., Jagodziński P.P. (2005). The Role of Mammalian DNA Methyltransferases in the Regulation of Gene Expression. Cell Mol. Biol. Lett..

[78] Szyf M. (2001). The Role of DNA Methyltransferase 1 in Growth Control. Front. Biosci..

[79] Wilson A.S., Power B.E., Molloy P.L. (2007). DNA Hypomethylation and Human Diseases. Biochim. Biophys. Acta.

[80] Cribbs A.P., Kennedy A., Penn H., Read J.E., Amjadi P., Green P., Syed K., Manka S.W., Brennan F.M., Gregory B. (2014). Treg Cell Function in Rheumatoid Arthritis Is Compromised by Ctla-4 Promoter Methylation Resulting in a Failure to Activate the Indoleamine 2,3-Dioxygenase Pathway. Arthritis Rheumatol..

[81] Cribbs A.P., Kennedy A., Penn H., Amjadi P., Green P., Read J.E., Brennan F., Gregory B., Williams R.O. (2015). Methotrexate Restores Regulatory T Cell Function Through Demethylation of the FoxP3 Upstream Enhancer in Patients With Rheumatoid Arthritis. Arthritis Rheumatol..

[82] Gaur N., Karouzakis E., Glück S., Bagdonas E., Jüngel A., Michel B.A., Gay R.E., Gay S., Frank-Bertoncelj M., Neidhart M. (2016). MicroRNAs Interfere with DNA Methylation in Rheumatoid Arthritis Synovial Fibroblasts. RMD Open.

[83] Fu L., Ma C., Cong B., Li S., Chen H., Zhang J. (2011). Hypomethylation of Proximal CpG Motif of Interleukin-10 Promoter Regulates Its Expression in Human Rheumatoid Arthritis. Acta Pharmacol. Sin..

[84] Neidhart M., Karouzakis E., Jüngel A., Gay R.E., Gay S. (2014). Inhibition of Spermidine/Spermine N1-Acetyltransferase Activity: A New Therapeutic Concept in Rheumatoid Arthritis. Arthritis Rheumatol..

[85] Wada T.T., Araki Y., Sato K., Aizaki Y., Yokota K., Kim Y.T., Oda H., Kurokawa R., Mimura T. (2014). Aberrant Histone Acetylation Contributes to Elevated Interleukin-6 Production in Rheumatoid Arthritis Synovial Fibroblasts. Biochem. Biophys. Res. Commun..

[86] Angiolilli C., Kabala P.A., Grabiec A.M., Rossato M., Lai W.S., Fossati G., Mascagni P., Steinkühler C., Blackshear P.J., Reedquist K.A. (2018). Control of Cytokine MRNA Degradation by the Histone Deacetylase Inhibitor ITF2357 in Rheumatoid Arthritis Fibroblast-like Synoviocytes: Beyond Transcriptional Regulation. Arthritis Res. Ther..

[87] Chen H., Pan J., Wang J.-D., Liao Q.-M., Xia X.-R. (2016). Suberoylanilide Hydroxamic Acid, an Inhibitor of Histone Deacetylase, Induces Apoptosis in Rheumatoid Arthritis Fibroblast-Like Synoviocytes. Inflammation.

[88] Nishida K., Komiyama T., Miyazawa S.-I., Shen Z.-N., Furumatsu T., Doi H., Yoshida A., Yamana J., Yamamura M., Ninomiya Y. (2004). Histone Deacetylase Inhibitor Suppression of Autoantibody-Mediated Arthritis in Mice via Regulation of P16INK4a and P21(WAF1/Cip1) Expression. Arthritis Rheum..

[89] Saouaf S.J., Li B., Zhang G., Shen Y., Furuuchi N., Hancock W.W., Greene M.I. (2009). Deacetylase Inhibition Increases Regulatory T Cell Function and Decreases Incidence and Severity of Collagen-Induced Arthritis. Exp. Mol. Pathol..

[90] Buhrmann C., Brockmueller A., Mueller A.-L., Shayan P., Shakibaei M. (2021). Curcumin Attenuates Environment-Derived Osteoarthritis by Sox9/NF-KB Signaling Axis. Int. J. Mol. Sci..

[91] Shakibaei M., Buhrmann C., Mobasheri A. (2011). Resveratrol-Mediated SIRT-1 Interactions with P300 Modulate Receptor Activator of NF-KappaB Ligand (RANKL) Activation of NF-KappaB Signaling and Inhibit Osteoclastogenesis in Bone-Derived Cells. J. Biol. Chem..

[92] Lomholt S., Mellemkjaer A., Iversen M.B., Pedersen S.B., Kragstrup T.W. (2018). Resveratrol Displays Anti-Inflammatory Properties in an Ex Vivo Model of Immune Mediated Inflammatory Arthritis. BMC Rheumatol..

