# Supplementary figures and images for: Epigenetic Regulation (Including Micro-RNAs, DNA Methylation and Histone Modifications) of Rheumatoid Arthritis: A Systematic Review
Source: Int J Mol Sci. 2021 Nov 10;22(22):12170. doi: 10.3390/ijms222212170 (PMC8625518; doi:10.3390/ijms222212170)

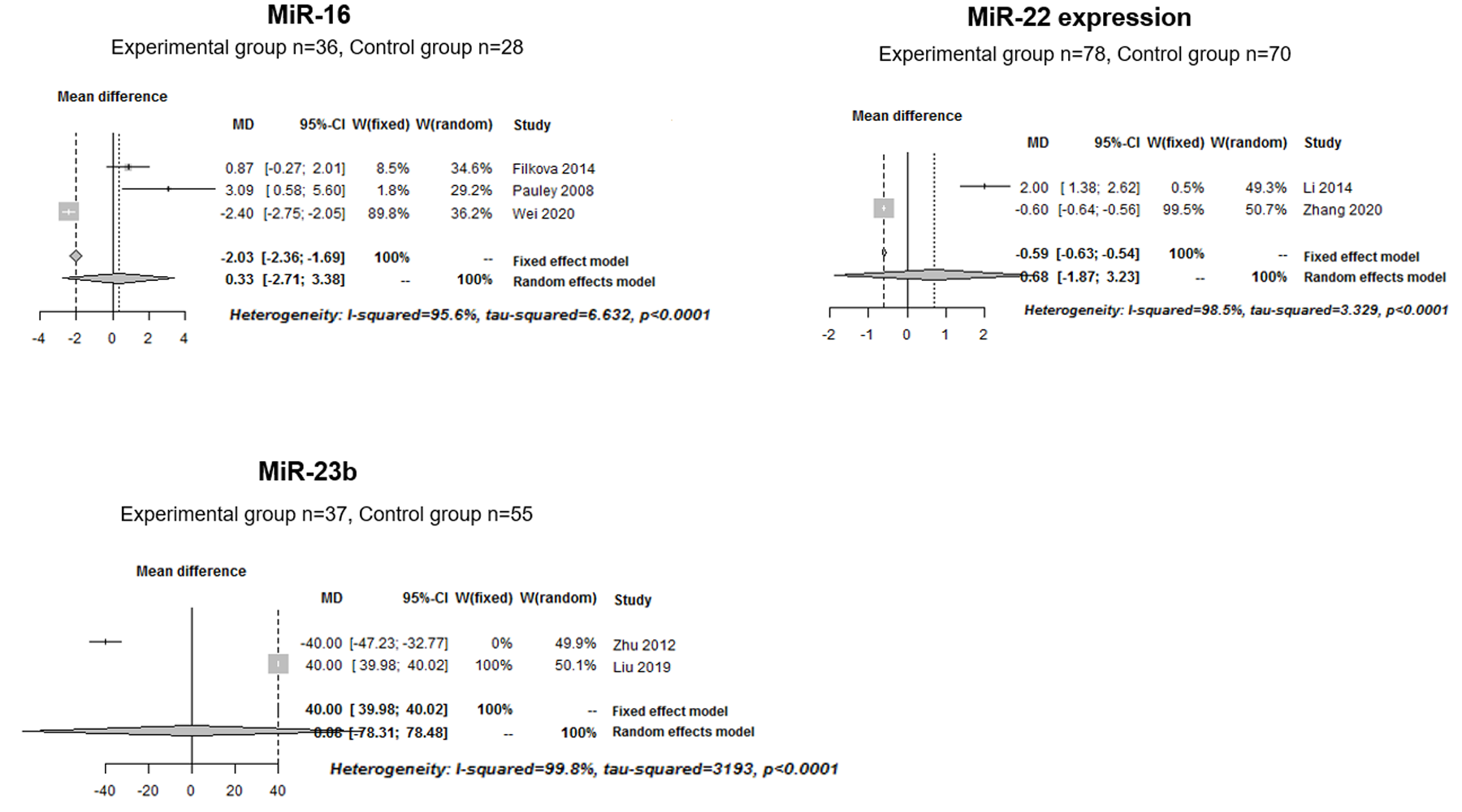

Supplement: Supplementary file 1 [file ijms-22-12170-s001.zip › Supplementary figure S1.tif]

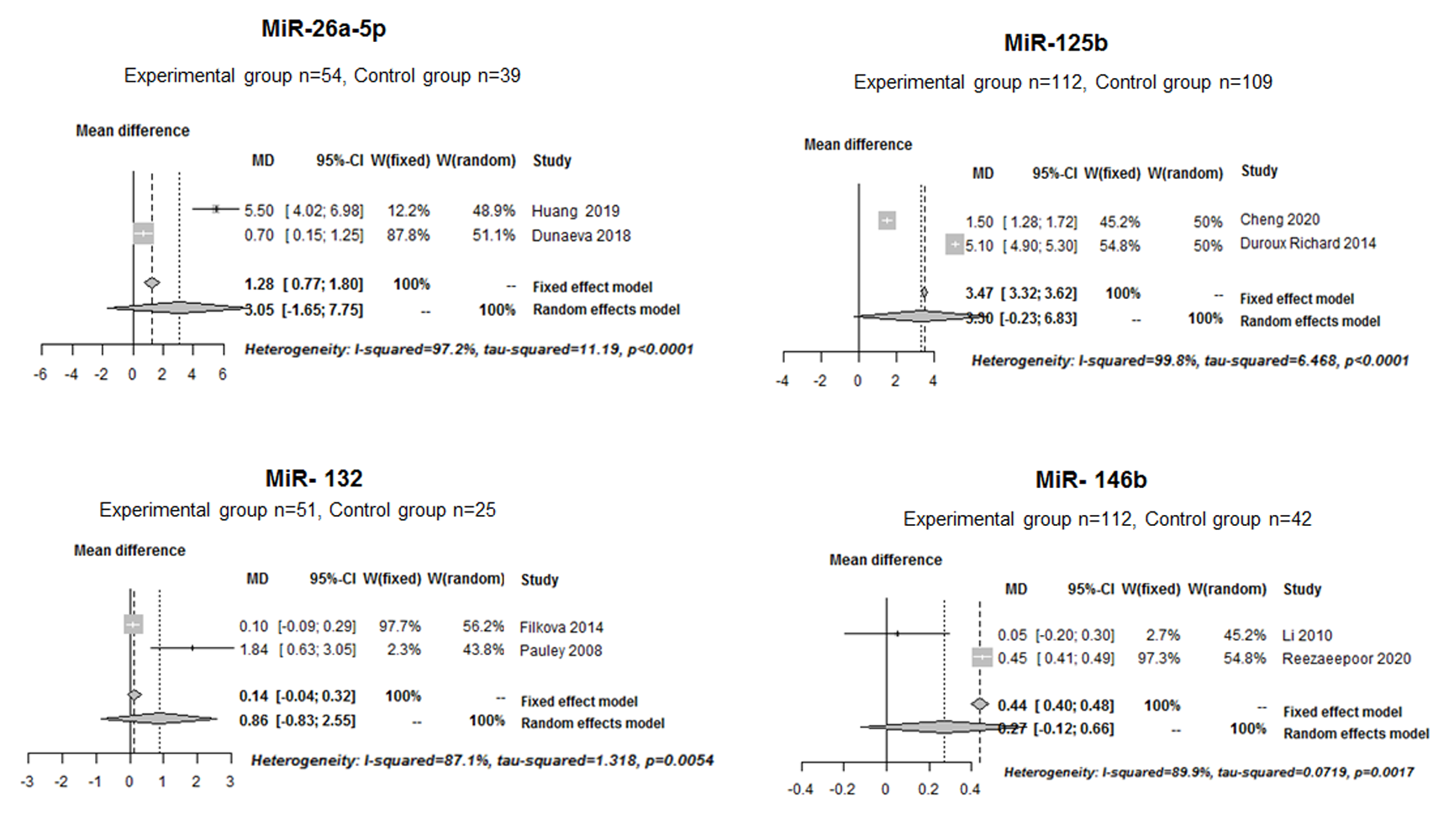

Supplement: Supplementary file 1 [file ijms-22-12170-s001.zip › Supplementary figure S2.tif]

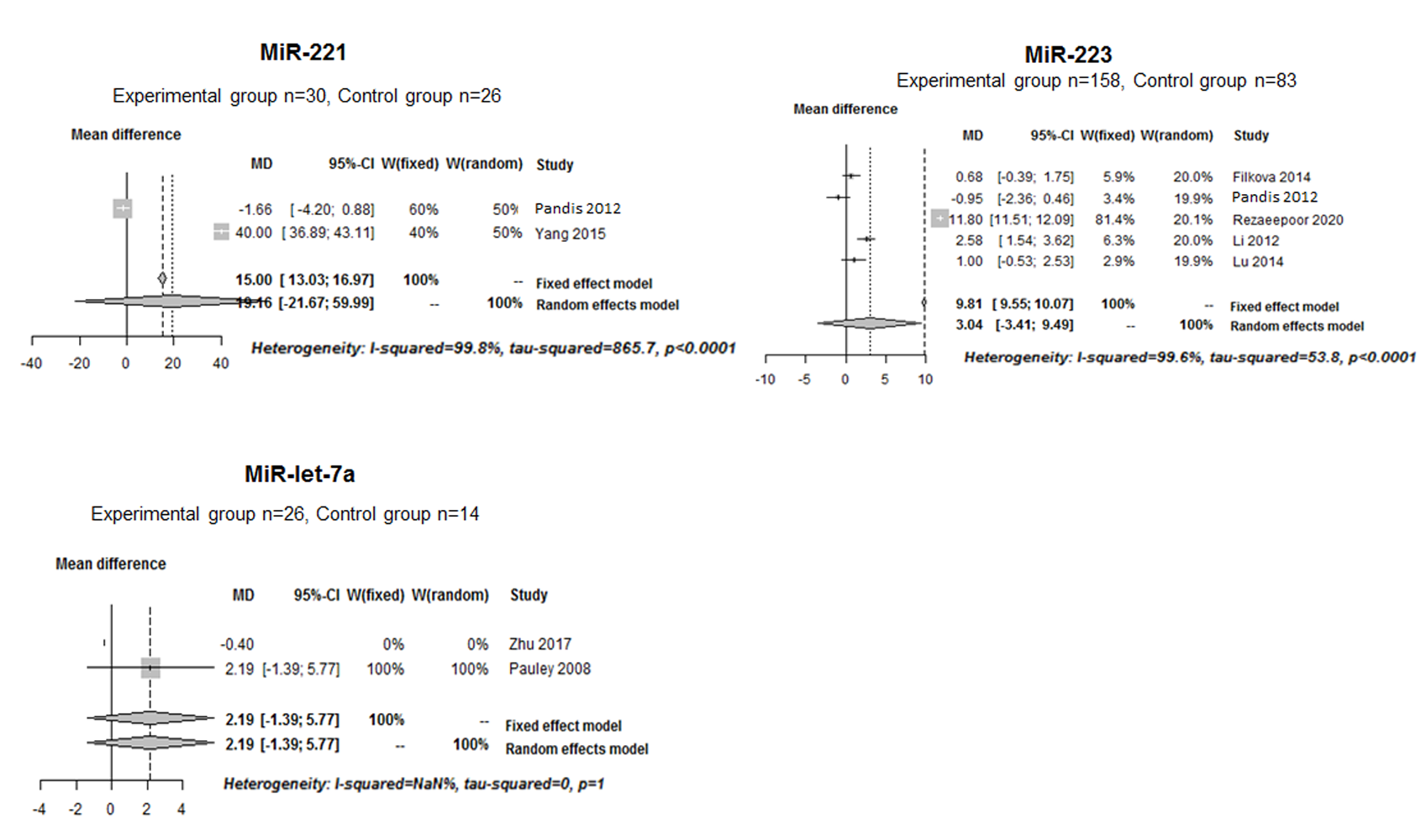

Supplement: Supplementary file 1 [file ijms-22-12170-s001.zip › Supplementary figure S3.tif]

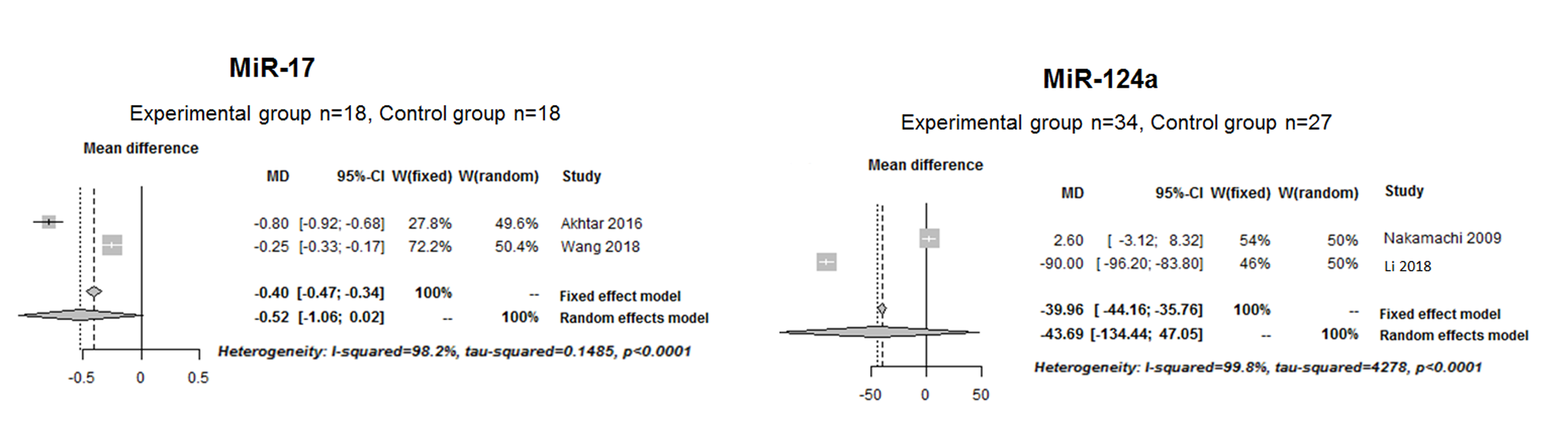

Supplement: Supplementary file 1 [file ijms-22-12170-s001.zip › Supplementary figure S4.tif]

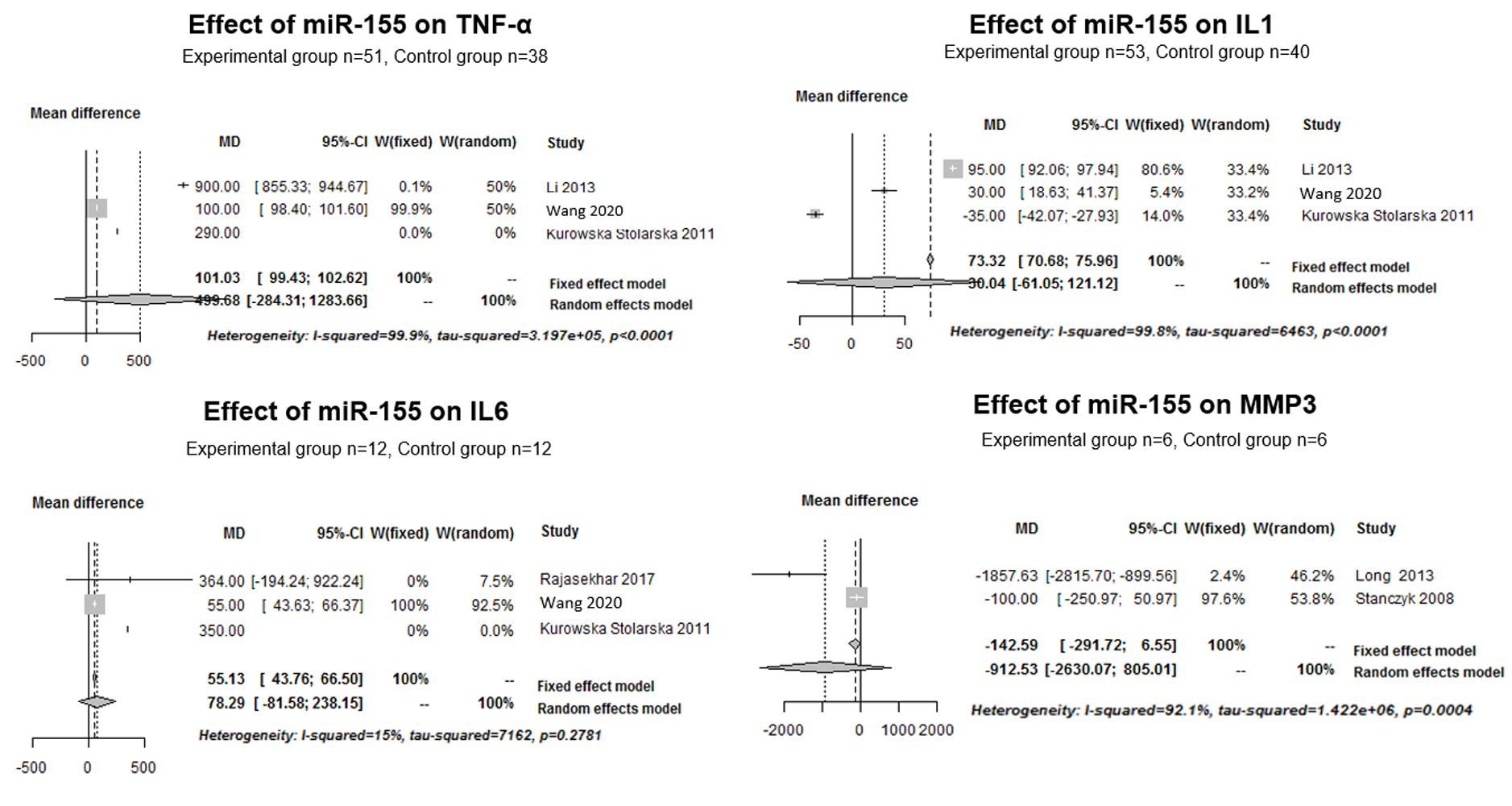

Supplement: Supplementary file 1 [file ijms-22-12170-s001.zip › Supplementary figure S5.tif]
